# Multi-exposure Laser Speckle Contrast Imaging (MECI)-Based Prediction of Blood Flow Using Random Forest (RF) With K-Means (KM)

**DOI:** 10.7759/cureus.40345

**Published:** 2023-06-12

**Authors:** Pankaj Jain, Saurabh Gupta

**Affiliations:** 1 Biomedical Engineering, National Institute of Technology, Raipur, IND

**Keywords:** clustering, machine learning, blood flow, laser speckle contrast imaging, multi exposure laser speckle contrast imaging

## Abstract

Blood flow prediction is very important for medical diagnosis, drug development, tissue engineering, and continuous monitoring. One commonly used method for studying blood flow is called multi-exposure laser speckle contrast imaging (MECI). It provides valuable insights into how blood flows through tissues and helps in diagnosing circulatory diseases. In our study, we used MECI to measure blood flow in real-time by taking multiple measurements with different exposure times and contrasts. To predict different blood flow rates ranging from 0.1 to 1 mm/s, we employed machine learning (ML) techniques like clustering and random forest (RF) or support vector machine (SVM) algorithms. The study showed that RF with K-means performance is found to be the most accurate technique for flow classification, with an accuracy of 98.5%, a precision of 92%, a specificity of 98.9%, and a classification error of 1.5%. Our study demonstrates that employing clustering and RF algorithms in MECI provides a robust and effective approach to predicting blood flow. This technique holds great potential for a wide range of applications in the medical and healthcare fields.

## Introduction

Blood flow is a vital process in the human body, referring to the movement of blood through the circulatory system. The human body relies heavily on blood flow to promote healthy cell growth, making it a fundamental biological process and also ensuring the proper functioning of organs and tissues. However, when blood flow is impaired, it can give rise to severe health conditions, including high blood pressure, cardiovascular disease, and even organ failure too [1]. To detect and diagnose such diseases early on, precise visualization and accurate measurement of blood flow are crucial. Hence, a diverse range of imaging systems comes into play, which is specially designed to determine the perfusion or effectiveness of blood flow [2].

Advancements in imaging technology and blood flow measurement techniques are essential to promote early detection and successful treatment of circulatory diseases. Various techniques, including Laser Doppler flow (LDF) meter, diffuse correlation spectroscopy, and diffuse correlation tomography, can be employed to measure perfusion in limited-layer and deep-layer tissues [3]. However, laser speckle contrast imaging (LSCI) stands out from the rest as it utilizes laser speckle to measure blood flow in tissue, providing imaging of vascular networks and associated hemodynamics [4].

Laser speckle contrast imaging is cost-effective, fast, and easy to use with basic optics, providing efficient real-time blood perfusion for imaging depths of up to 1 mm, where speckle pattern gives information about microvascular blood flow [5-6]. However, conventional LSCI systems have limitations in sensitivity and accuracy due to their reliance on a single exposure time [7].

To overcome these limitations, multi-exposure laser speckle contrast imaging (MECI) extends LSCI by capturing different exposure times with high-speed cameras, allowing for more accurate and sensitive speckle contrast measurements [8]. MECI provides real-time quantitative measurements and offers a deeper understanding of blood flow dynamics and perfusion in biological systems [9].

Multi-exposure laser speckle contrast imaging has gained increased attention from researchers when combined with other imaging techniques to create a more stable instrument. Briers et al. highlighted the limitations of single-exposure LSCI and proposed that MECI can solve many problems [10]. Parthasarathy et al. developed a framework for MECI data that accounted for the effect of stationary scatters on speckle contrast, improving its accuracy [8]. Machine learning (ML) algorithms with LSCI/MECI have shown promise in advancing perfusion data analysis and interpretation.

Machine learning has proven itself as a powerful tool in diverse domains by leveraging past data to make predictions without explicit instructions. In the medical field, ML algorithms have made notable strides in disease diagnosis, treatment planning, and drug development, revolutionizing healthcare practices. In LSCI, researchers such as Basak utilized support vector machines (SVMs) to monitor wound progression with high sensitivity and specificity [11]. Keilbach et al. developed ML-based methods for fingerprint presentation attack detection, achieving low error rates, while Kolberg et al. further improved upon these results [12-13]. Hao demonstrated the feasibility of using a three-dimensional convolutional neural network for quantitative blood perfusion measurement in LSCI [14]. In MECI, Fredriksson et al. leveraged ML algorithms to calculate precise perfusion estimates, and Stebakov et al. proposed an ML technique for recognizing physiological fluid flow rates [15-16]. Hultman introduced an artificial neural network for speed-resolved perfusion imaging, and Jain presented a generative adversarial network for the reliable prediction of blood flows in diverse scenarios [3, 17].

Our study suggests that while there have been several studies on the application of ML in LSCI and MECI, there is a lack of research on blood flow prediction specifically in MECI. This motivated us to conduct our research to explore the potential of ML with MECI for flow prediction. We employed various classification strategies, including random forest (RF), K-means (KM), support vector machine (SVM), RF with K-means (RF-KM), and support vector machine with K-means (SVM-KM), to develop ML models using multi-class conventional classification methods. We then evaluated the performance of our proposed method using several metrics, including accuracy, precision, recall, specificity, and error rate, to optimize our customized MECI system. This proof-of-concept analysis aims to contribute to developing more accurate and efficient blood flow prediction models using ML with MECI, contributing to enhanced perfusion analysis, and personalized medical diagnostics, leading to improved clinical applications and a deeper understanding of biological systems.

## Materials and methods

**Figure 1:**
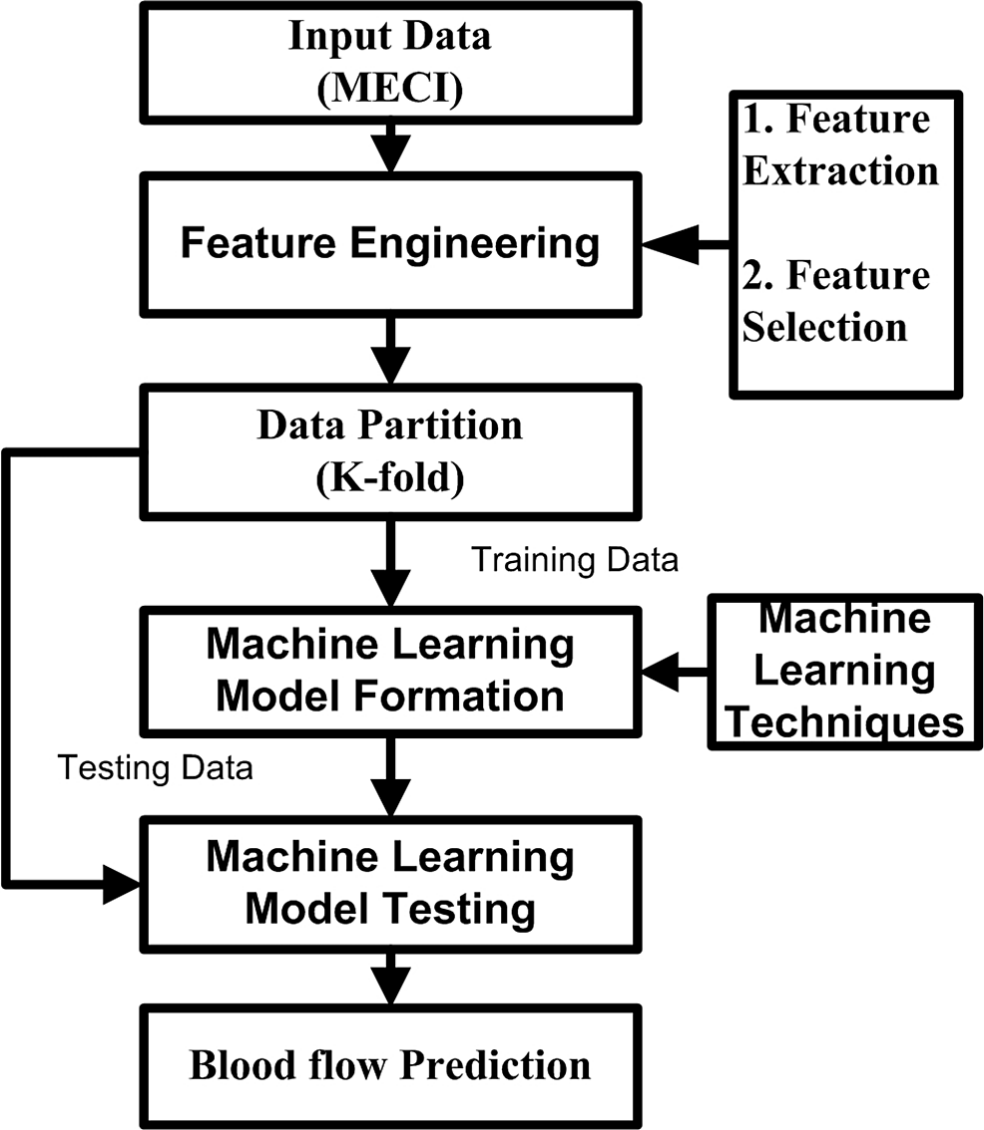
Process flow of machine learning techniques in our study.

The methodology of our work involves several steps. First, the data are collected and pre-processed to ensure it is clean and relevant. Then, selecting important features that will help the model learn and predict accurately. Then, the data are split into training and testing sets. The ML algorithms are chosen and trained using the training set. After training, the performance of the algorithm is evaluated on the testing set. If the performance is not satisfactory, the algorithm is fine-tuned and retrained. Once the performance is acceptable, the algorithm can be deployed and used for predictions on new data [18]. Figure 1 shows the process flow of our work.

Experimental dataset

This research uses the dataset collected from the IIT Mumbai bioinformatics lab. The study made use of a flow phantom to simulate blood flow in the body, which was created by using a diluted intralipid solution (Fresenius Kabi intralipid 20%) and controlling it using a syringe pump (New era system, Farmingdale, NY). The flow phantom was illuminated with a uniform 785 nm laser source (L785P090, Thorlabs Inc., Newton, NJ) using a diffuser in the region of interest (ROI) in the phantom. The multi-exposure intensity images were then captured using a camera (Basler acA 640-120 µm, Thorlabs Inc., Newton, NJ) with an appropriate f-number to match the speckle-to-pixel ratio. This allows the researchers to have a controlled environment to test the performance of the classifier models as well as to have a standard dataset for future research. The original database consists of 50,000 images of MECI. The database split into 500 images with 10 exposures (100-3981) μ-s of each 10 flows (between 0.1 and 1 mm/s) [3].

Feature engineering

Feature extraction is a vital step in ML that can help improve the performance of algorithms and make the data more manageable. In the context of MECI, the speckle contrast (K) is a significant measure of speckle decorrelation related to the scatterer’s motion. A low K value indicates motion, whereas a high K value indicates no motion in the ROI.

In this study, several feature extraction techniques are employed to extract relevant features from MECI images, including statistical, texture, gray-level co-occurrence texture, gray-level run-length texture, local binary, and scale invariant feature transform features. These features serve as inputs for ML algorithms to classify flow in the system. The use of multiple feature extraction techniques increases the robustness of the results and enhances the overall performance of the system [19].

The study utilized principle component analysis (PCA) to select the most significant features. These significant features were then divided into two datasets using K-fold cross-validation (K=10), with one dataset used for model training and the other for testing the ML model. The training dataset was used to train the model, while the testing dataset was used to evaluate the performance of the trained model [20].

Machine learning

Machine learning has emerged as a promising tool in various fields, due to its ability to learn from past data and make predictions without explicit instructions. The use of ML algorithms in medical applications has led to significant advancements in areas such as disease diagnosis, treatment planning, and drug development.

The ML techniques can be broadly classified into two categories: supervised learning (SL) and unsupervised learning (UL). In our study, we compared both SL- and UL-based algorithms [19]. RF is an SL algorithm that is an ensemble method that builds multiple decision trees on the input data and then combines them to make a final prediction. It is an empirical approach that is better than the standard decision tree by reducing overfitting. The basic idea behind RF is to construct multiple decision trees on a random subset of the input data and features. Then, the final prediction is made by aggregating the predictions of these decision trees. RF is a powerful algorithm that can handle high-dimensional and complex data, and it is often used for classification and regression tasks. SVM is an SL technique that classifies the data by a hyper-plane separation which is decided by the maximum distances between the closest data point of either class. The hyperplane separation is done by tuning the kernel which transforms the input data into a linear, polynomial, radial basic function, and many more [15, 21].

Unsupervised learning methods like clustering can be very useful for identifying patterns and relationships within large datasets, particularly when there are no pre-defined classes or training examples available. Clustering algorithms like K-means (KM) are particularly well-suited for this task, as they can group similar data points based on their characteristics and assign them to different categories or clusters. This can be very useful for identifying trends within the data, as well as for identifying outliers or anomalies that may require further investigation [21].

Clustering and other methods can help to combine logging responses for classification, which can be particularly useful for analyzing different types of data and identifying patterns. By analyzing the relationships between logging responses and blood flow, it is possible to gain insights into the behavior of different systems or applications, identify potential issues or vulnerabilities, and improve overall system performance and reliability. So, the modeling and forecasting process using RF-KM or SVM-KM will be a robust and effective technique for predicting blood flow. The use of KM clustering to divide the data into categories helps to address the issue of poor relationship between logging response and blood flow, by grouping similar data points based on their characteristics. The RF or SVM algorithm is then applied to each category separately, allowing for a more accurate prediction of blood flow based on the specific features of each group [22].

Performance analysis

Evaluating the performance of different classifiers on the same dataset, as well as the performance of the same classifier on different datasets, can provide valuable information for improving the accuracy of the classifier. Overall, the use of a confusion matrix and associated performance metrics can greatly enhance the effectiveness of machine learning models [23].

A confusion matrix is a useful tool for evaluating the performance of a classifier in predicting outcomes based on test data with known true values. Various performance metrics obtained from the confusion matrix are shown in Table 1 including accuracy, precision, recall, specificity, F1-score, and classification error rate, which provide valuable insights into the classifier's accuracy.

**Table 1:** Evaluation metrics of our predictive model. tpP, true positive; tnN, true negative; fpP, false positive; fnN, false negative

Metrics	Methods
Accuracy	$\frac{tpP+tnN}{tpP+tnN+fpP+fnN}$
Recall	$\frac{tpP}{tpP+fnN}$
Precision	$\frac{tpP}{tpP+fpP}$
Specificity	$\frac{tnN}{tnN+fpP}$
F1-score	$\frac{2 \times Precision \times Recall}{Precesion+Recall}$​​​​​​​
Classification error rate	$1-Accuracy$​​​​​​​

## Results

In our work, models were created using TensorFlow 2.0.0 in Python 3.6.10 and were run on a system with an Intel Xeon e-5 2630V3 @ 2.2 GHz processor, 64 GB RAM, and a QuadroM5000 GPU. In the study, 50000 MECI data were labeled with 10 classes, and the efficacy of each machine learning model is determined by performing a series of experiments on the MECI data for predicting different perfusion in the range (0.1-1 μm/s) with a given set of 10 different exposure times.

**Figure 2:**
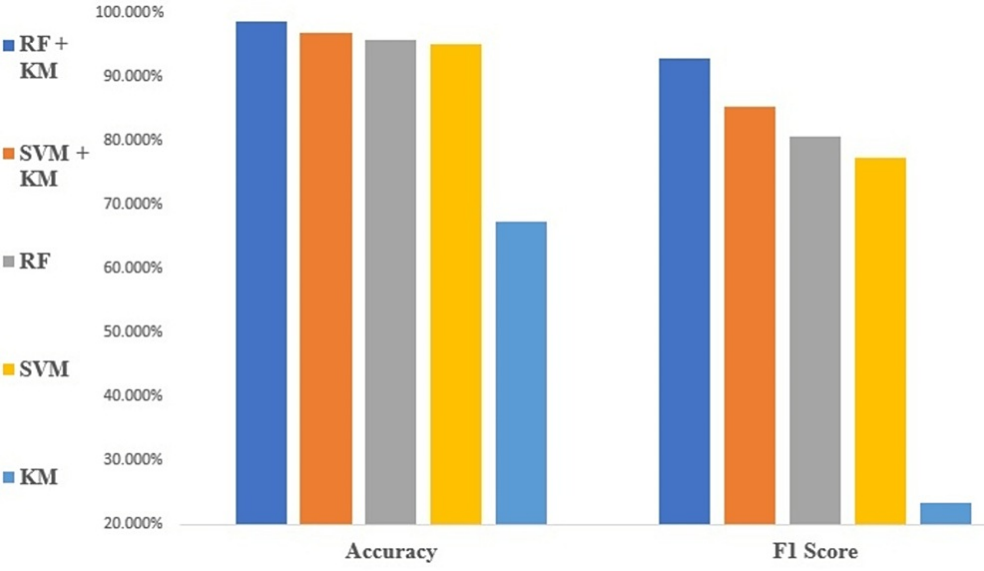
Accuracy and F1-score performance of different flows for all the classifiers.

**Figure 3:**
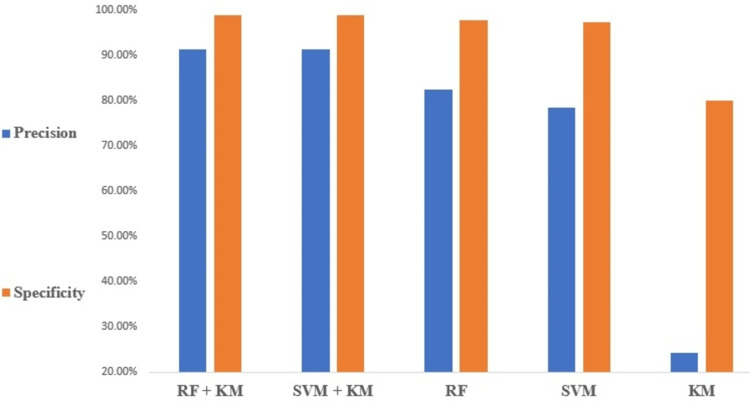
Specificity and precision performance of the different ML models. ML, machine learning

**Figure 4:**
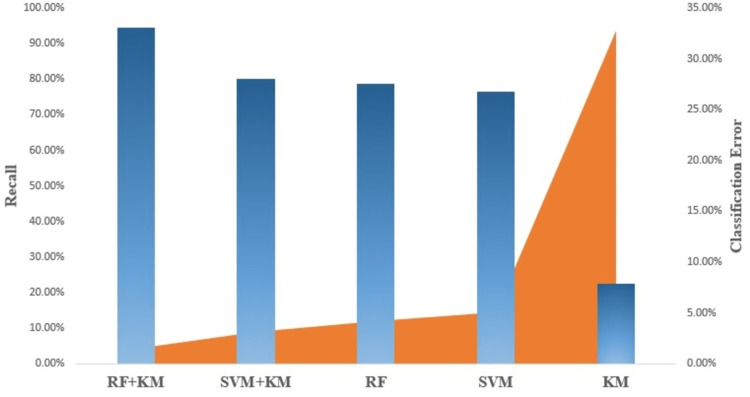
Recall and classification error performances of the different ML models. ML, machine learning

Figure 2 compares the accuracy performance and F1-score of different flows for all the classifiers. A comparison of the specificity and precision performances of the different ML models is shown in Figure 3. The other performance parameter recall and classification errors are shown in Figure 4.

## Discussion

In the field of medical diagnosis, the prediction of blood flow is a crucial factor. In our study, the focus is on evaluating the performance of five different ML-based algorithms (RF, KM, SVM, RF-KM, and SVM-KM). The algorithms were tested with varying flows of blood and different exposure times. The study aimed to determine the accuracy, F1-score, precision, recall, specificity, and classification error of these different algorithms when applied to the complete dataset. The evaluation metrics were used to determine the performance of each algorithm on the given dataset. The study intended to select the best-performing algorithm that could predict blood flow accurately and with a high degree of confidence.

Note on ML techniques

The RF can handle missing data and outliers in the dataset and also has the advantage of handling high-dimensional data and can capture nonlinear relationships between the features and the outcome, whereas SVM requires careful feature selection and pre-processing to achieve good performance. It is also sensitive to the choice of kernel function and parameters. KM, on the other hand, is not specifically designed for classification tasks and may not be suitable for datasets with high-dimensional features. It requires the number of clusters to be specified in advance, which may be challenging for datasets with unknown or varying numbers of classes [20, 23].

The RF-KM and SVM-KM algorithms have superior performance due to their ability to effectively handle high-dimensional datasets and capture complex patterns in the data. The combination of KM clustering with RF and SVM algorithms allowed for more accurate feature extraction and improved classification of the blood flow patterns in MECI images. KM clustering helps to reduce the dimensionality of the feature space, making it easier for the algorithms to identify relevant features and relationships in the data. Additionally, RF and SVM algorithms are both known for their ability to handle high-dimensional data and effectively classify complex patterns in the data. RF-KM uses both clustering and ensemble techniques to improve the robustness and accuracy of the model. Similarly, SVM-KM uses clustering to reduce the dimensionality of the data and improve the efficiency of the SVM model. This makes them particularly suitable for the analysis of MECI images with high flow velocities and complex patterns.

Note on performance metrics

The performance of the models is shown in Figures 2-4 as indicated by their accuracy and F1-score, precision and specificity, and recall and classification error rate. The model is very reliable in predicting blood flow values, with a low likelihood of false positives or false negatives. The accuracy of the model is impressive, with a high level of agreement between predicted and actual values. The precision and recall values show that the model can identify relevant instances and avoid false positives. The specificity value indicates that the model is very good at identifying negative instances, also F1-score value shows effectiveness at identifying both true positives and true negatives instances, while the low classification error rate suggests that the model is highly robust and able to avoid misclassifying data points [23].

Furthermore, RF-KM and SVM-KM algorithms have shown to have better performance metrics, including accuracy, f1-score, precision, specificity, and recall, compared to other models, which indicates that both algorithms can cluster data effectively, allowing for improved accuracy and precision in prediction. Additionally, these algorithms can avoid overfitting and increase the generalization ability of the model, also both can avoid false positives and false negatives and make better predictions. We have observed that the KM model shows poor prediction results, which is reflected in the performance results. The poor performance of the KM model can be attributed to its inability to effectively cluster the data. This leads to inaccurate categorization of data points, resulting in poor prediction results and indicating that the model is less effective in predicting blood flow values. Hence this model can be used as a benchmark for other models. In summary, RF-KM can handle high dimensional data, reduce overfitting and provide better results, making it a better choice for MECI data compared to other classification models.

Note on ML performance

The accuracy of blood flow prediction is influenced by various factors, including imaging system quality, imaging parameters, and underlying physiology. In this study, it was observed that the RF-KM algorithm achieved the highest accuracy of 98.89% for all flow rates, indicating its superior performance in predicting blood flow in MECI images. The SVM-KM algorithm also showed high accuracy, achieving almost similar results of 98%. These high accuracy results may be due to the prominent features present in the MECI data that are easily identifiable by all the algorithms tested. These findings highlight the importance of selecting appropriate algorithms for the specific task and dataset and the potential benefits of using feature extraction techniques such as KM in improving the accuracy of blood flow prediction.

Note on benchmark comparison

Table 2 compares different studies focusing on the application of ML algorithms in LSCI and MECI. These studies employ various ML techniques such as convolutional neural networks, artificial neural networks, and generative adversarial networks to address the challenges associated with measuring blood flow using MECI.

**Table 2:** A benchmark table of the proposed method with the previous study. CNN, convolution neural network; ANN, artificial neural network; GAN, generative adversarial network; RF, random forest; KM, K-Means; SVM, support vector machine; LSCI, laser speckle contrast imaging; MECI, multi-exposure laser speckle contrast imaging; LDF, Laser Doppler flow;

Author	Methods used	Key findings	Output
Hao et al. [14]	Three dimensional – CNN, behavioral feature learning	Developed CNN-LSCI for quantitative blood perfusion measurement in LSCI with improved accuracy compared to traditional LSCI and MECI.	LSCI velocities prediction model (CNN-LSCI) with 0.33 MSE and 0.34 MAPE, verified by 10 phantom velocities with a correlation of 0.98.
Fredriksson et al. [15]	ANN	Utilized ANN to process MECI data and calculate perfusion estimates resembling LDF perfusion with high accuracy.	High accuracy perfusion estimates comparable to LDF perfusion, correlation coefficients of 1.000 (noise-free), 0.993 (moderate noise), and 0.995 (in vivo data).
Stebakov et al. [16]	Feed-forward ANN	Combined speckle contrast imaging with a feed-forward artificial neural network to recognize physiological fluids flow rate, improving recognition accuracy.	Increased fluid flow rate recognition accuracy from about 65%-89%
Jain et al. [3]	GAN	Utilized GAN for reliable prediction of blood flows in diverse scenarios in MECI.	Accuracy 98%, recall 93%, specificity 99%, and classification error 2%
Proposed method	RF, KM, SVM, RF with KM, and SVM with KM	Employing clustering and ML algorithms in MECI provides a robust and effective approach to predicting blood flow.	Accuracy 98.5%, F1- score 93%, recall 94.5%, specificity 98.5%, precision 92%, and classification error 1.5%

Our research contributes to the field by evaluating and comparing the performance of different ML techniques in predicting blood flow values. By doing so, we aim to improve the accuracy and reliability of MECI systems for measuring blood flow. The application of ML techniques to real-time MECI data is a novel approach and can provide insights into the use of ML for predicting blood flow values in real time. The comparison of different ML techniques can also aid in selecting the most effective algorithm for the prediction of blood flow values, which can lead to better accuracy and reliability in MECI systems. Therefore, our study can have significant implications for the development of advanced MECI systems for the measurement of blood flow. This advancement will not only enhance medical diagnostics but also deepen our understanding of biological systems and their complex dynamics. Ultimately, this study paves the way for enhanced clinical practices and advancements in the field of biomedical research.

## Conclusions

In conclusion, this study has shown that ML techniques can be effectively applied to real-time MECI data for the prediction of flow in the MECI system. The comparative assessment of RF, KM, SVM, RF with KM, and SVM with KM showed that RF with KM is the most accurate technique for flow classification, with high values for accuracy, F1-score, precision, recall, and specificity. The study's stability and reliability were further supported by performance evaluation.

This study contributes to the field of MECI systems by providing insights into the application of ML techniques for improving the accuracy and reliability of flow measurement. The findings of this study can be applied to the development of better MECI systems, which are important for the diagnosis and treatment of various medical conditions. Future work should focus on exploring the potential of deep learning techniques for flow classification, which requires a larger training dataset to fully harness the capability of deep learning. Overall, this study highlights the potential of ML techniques for improving the accuracy and reliability of MECI systems and paves the way for future research in this field.
